# Special Issue: Thermal Analysis of Materials

**DOI:** 10.3390/ma14174923

**Published:** 2021-08-30

**Authors:** Sergey V. Ushakov, Shmuel Hayun

**Affiliations:** 1 School of Molecular Sciences and Center for Materials of the Universe, Arizona State University, Tempe, AZ 85287, USA; 2 Department of Materials Engineering at the Ben-Gurion University of the Negev, Beer-Sheva 84105, Israel

The measurement of any physical property as a function of temperature brings the method used into the realm of thermal analysis. This makes “Thermal Analysis of Materials” a broad interdisciplinary subject. This Special Issue combines contributions solicited by editors that reflect their scientific interests and networks, with general related submissions received over two years. It contains 16 articles and 1 short review submitted by authors from 10 countries.

In several papers, well-established techniques of differential thermal analysis, thermogravimetry, and gas adsorption calorimetry are applied to research related to environmental problems, such as capturing CO_2_ from industrial processes by calcium looping [1]; the utilization of copper tailing wastes as an addition to Portland cement [2]; the reuse of soda lime CO_2_ absorbent in spacecrafts, submarines, anesthetics, and diving apparatuses [3]; and the passive fire protection offered by inorganic material-based insulation [4].

Another set of papers deals with the development of new or application-specific approaches, namely laser interferometry techniques for high precision measurements on non-standard samples, such as composite truss structures [5]; measurements of high temperature compressive creep in spark plasma sintering apparatuses [6]; measurements of the density of liquid oxides with aero-acoustic levitators [7]; and the simultaneous estimation of thermal conductivity and heat capacity in metal alloys [8].The range of materials covered includes polymers [9,10]; natural complex inorganic materials, such as natural sorbents [1] and industrial-grade thermal insulation [4]; single phase ceramic materials, such as spinel MgAl_2_O_4_ [11], rare earth sesquioxides [12], and double perovskite cobaltites [13]; metals, alloys, and intermetallic compounds [8,14,15,16].

The use of thermal analysis techniques to elucidate phase transformation kinetics can be found in papers on the hydration of cement [2] and in the study of microstructural evolutions in high-carbon and chromium-bearing steel [15]. A review by Plota and Masek [10] is devoted to the extrapolation of kinetic data on polymers’ degradation for lifetime predictions. A paper by Frock et al. [16] provides a new method to study kinetics at heating rates between 5 and 1000 K per second. These heating rates are relevant to welding and laser melting processing but too fast for conventional differential scanning calorimeters and too slow for fast-scanning chip calorimetry. Examples of the applications of thermal analysis and calorimetry techniques to obtain thermodynamic data vary from measurements of enthalpies of water adsorption on defect spinel surfaces [11] to phase transformation enthalpies and entropies above 2000 °C in rare earth oxides [12].

Thermal analysis contributes to the calculation of phase diagrams (CALPHAD), both through experimental phase diagrams and by providing thermodynamic data for optimizations. Krishon et al. [17] extend the CALPHAD approach by calculating pressure dependence of several binary metal phase diagrams based on the temperature dependence of sound velocity and density data in liquid alloys. Thus, this Special Issue provides a sampling of the current use, diversity, and ongoing developments of techniques and approaches in the thermal analysis of materials.
